# A Right Atrial Mass with Fever and Cutaneous Nodules

**DOI:** 10.14797/mdcvj.1126

**Published:** 2022-07-11

**Authors:** Feng Gao, Stephanie Koh, Sara Taveras-Alam, Umair Khalid

**Affiliations:** 1 Baylor College of Medicine, Houston, Texas, US; 2 Michael E. DeBakey VA Medical Center and Baylor College of Medicine, Houston, Texas, US

**Keywords:** extranodal lymphoma, cardiac magnetic resonance imaging, multimodality imaging, anaplastic large cell lymphoma, primary cardiac tumor

## Abstract

Primary cardiac tumors, although exceedingly rare, should be considered in the differential diagnosis during workup of any cardiac mass. Extranodal cardiac lymphomas have a natural aggressive course due to delayed diagnosis. We present a 71-year-old male with a dual-chamber pacemaker who presented with fevers and new cutaneous nodules. He was found to have a right atrial primary anaplastic large-cell lymphoma and had a complete metabolic response after chemotherapy. Our case highlights the importance of a multimodality approach in the diagnosis of cardiac tumors and during follow-up after treatment.

## Introduction

The differential diagnosis for a right atrial mass includes primary tumors, metastases, vegetations, and thrombus. Cancer metastases, by far, are the most common cause of cardiac tumors, with melanoma, breast, lung, and esophageal carcinomas leading the list.^1^ Cardiac lymphomas make up less than 2% of primary cardiac tumors.^1^ Anaplastic large-cell lymphoma (ALCL) accounts for 3% of adult cases of non-Hodgkin’s lymphoma, and only case reports of cardiac involvement have been described.^2,3,4,5^ This poorly differentiated lymphoma is classically diagnosed by CD30 positive staining and lack of B-cell (CD20) and T-cell markers (CD3, CD4, CD8).^2^ Prognosis of primary cardiac lymphoma is generally poor due to delayed diagnosis and aggressive disease course.

## Case Presentation

**Figure 1:**
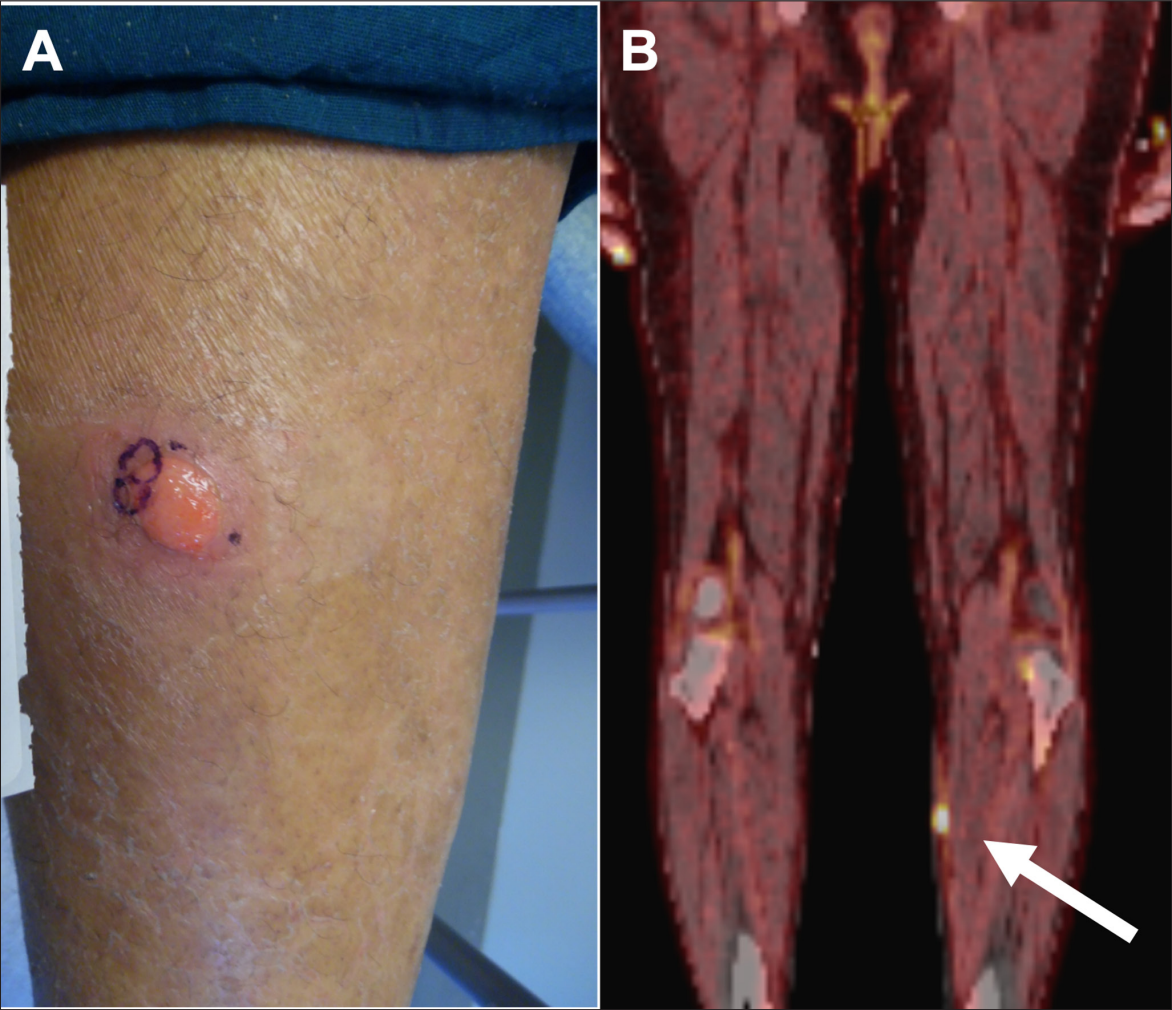
**(A)** Friable erythematous nodule of left medial leg. **(B)** Sagittal positron emission tomography with computed tomography with avid uptake in the same nodule of left leg (arrow).

**Figure 2:**
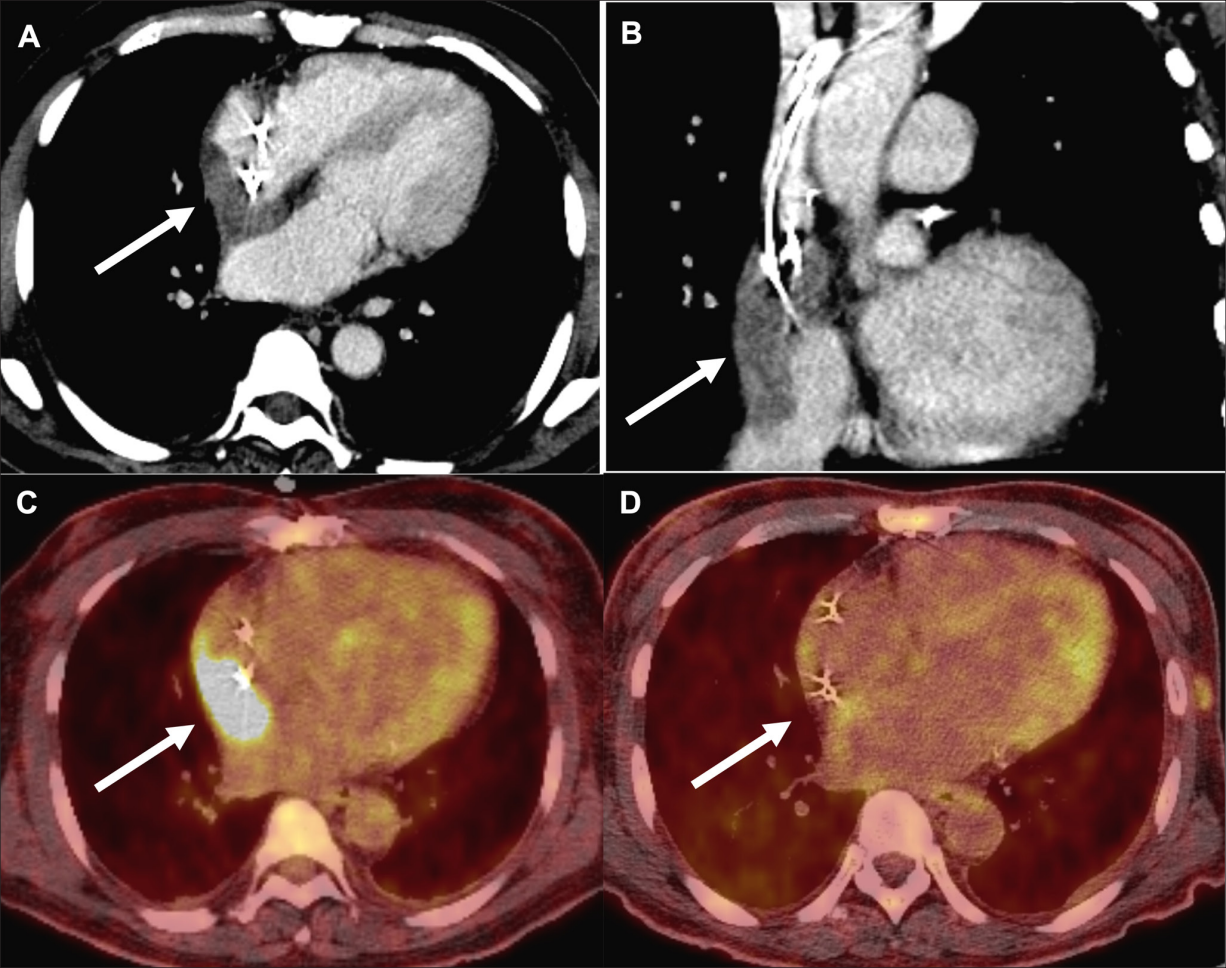
**(A)** Mass-like right atrial filling defect (arrow) demonstrated on transverse contrast computed tomography (CT). **(B)** Right atrial mass (arrow) redemonstrated on sagittal contrast CT encasing pacemaker leads. **(C)** Prechemotherapy positron emission tomography with computed tomography (PET-CT) demonstrating avid uptake of a right atrial mass (arrow) encasing pacemaker leads in transverse view. **(D)** PET-CT demonstrating complete metabolic response (arrow) post-treatment in transverse view.

A 71-year-old immunocompetent male with a dual-chamber pacemaker presented with 2 months of fevers, chills, weight loss, and new cutaneous nodules (Figure 1 A). Initial complete blood count showed a normal leukocyte count. Infectious workup, including blood cultures, was unremarkable. A contrasted computed tomography (CT) of the chest found a filling defect in the right atrium extending into the superior vena cava, which was concerning for thrombus (Figure 2 A, B). A fluorodeoxyglucose (18F-FDG) positron emission tomography with computed tomography (PET-CT) revealed a region of avid uptake in the right atrium adjacent to pacemaker leads, suggestive of a possible infected thrombus (Figure 2 C). Notably, avid uptake on multiple cutaneous lesions was also observed, suggestive of possible cutaneous lymphoma (Figure 1 B). Biopsies of skin lesions returned CD30-positive cells with immunohistochemistry staining negative for CD4 and CD8. At the time, it was thought that this was likely either a localized cutaneous ALCL—given lack of systemic uptake on 18F-FDG PET-CT scan—or lymphomatoid papulosis, a benign variant of primary cutaneous ALCL.

**Figure 3:**
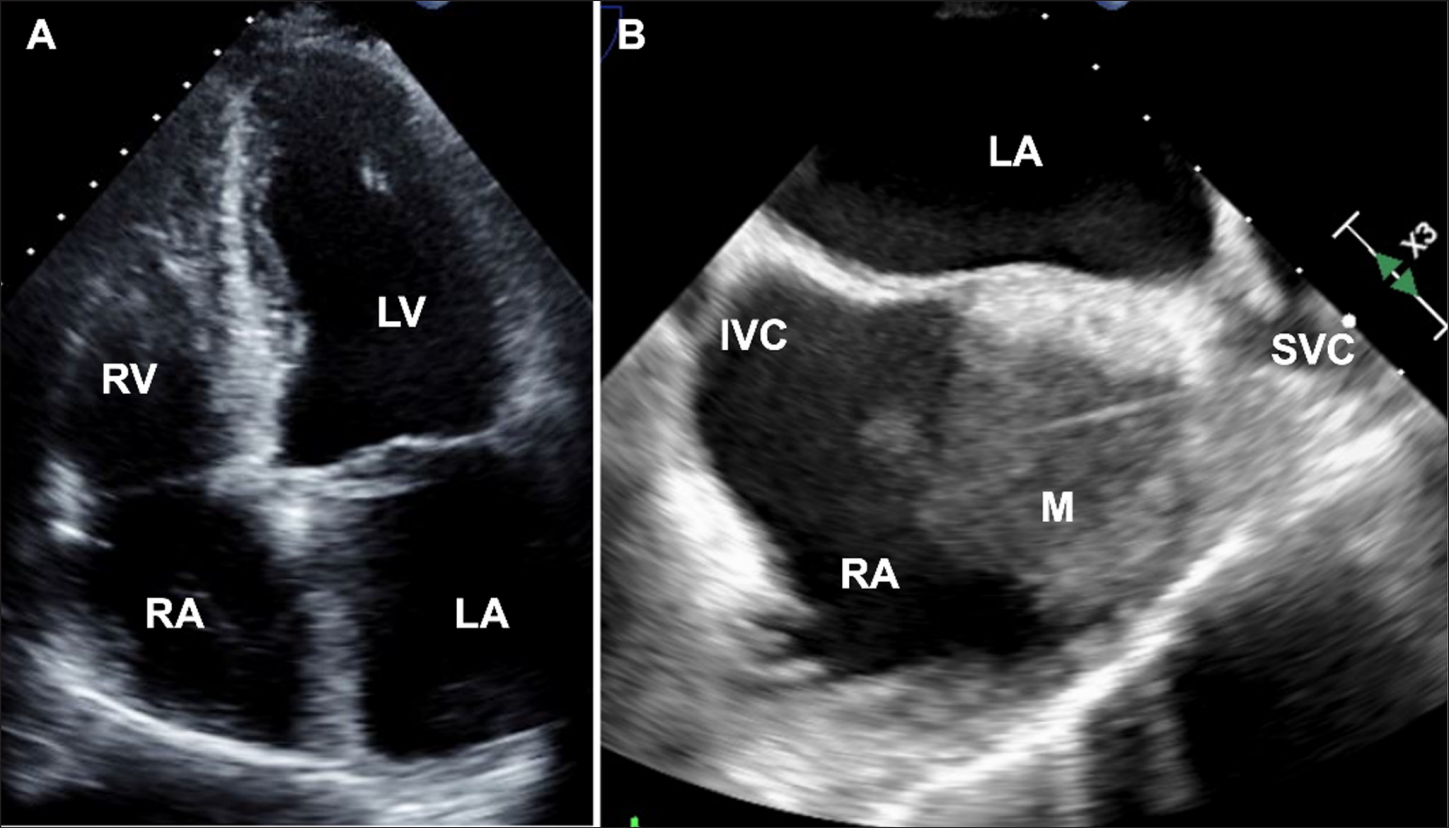
**(A)** Apical four-chamber view on transthoracic echocardiogram did not show any evidence of mass or thrombus in the RA. **(B)** Mid-esophageal bicaval view on transesophageal echocardiogram shows the mass extending from the superior vena cava into the RA along the device lead. RA: right atrium; LA: left atrium; IVC: inferior vena cava; M: mass; SVC: superior vena cava

**Figure 4:**
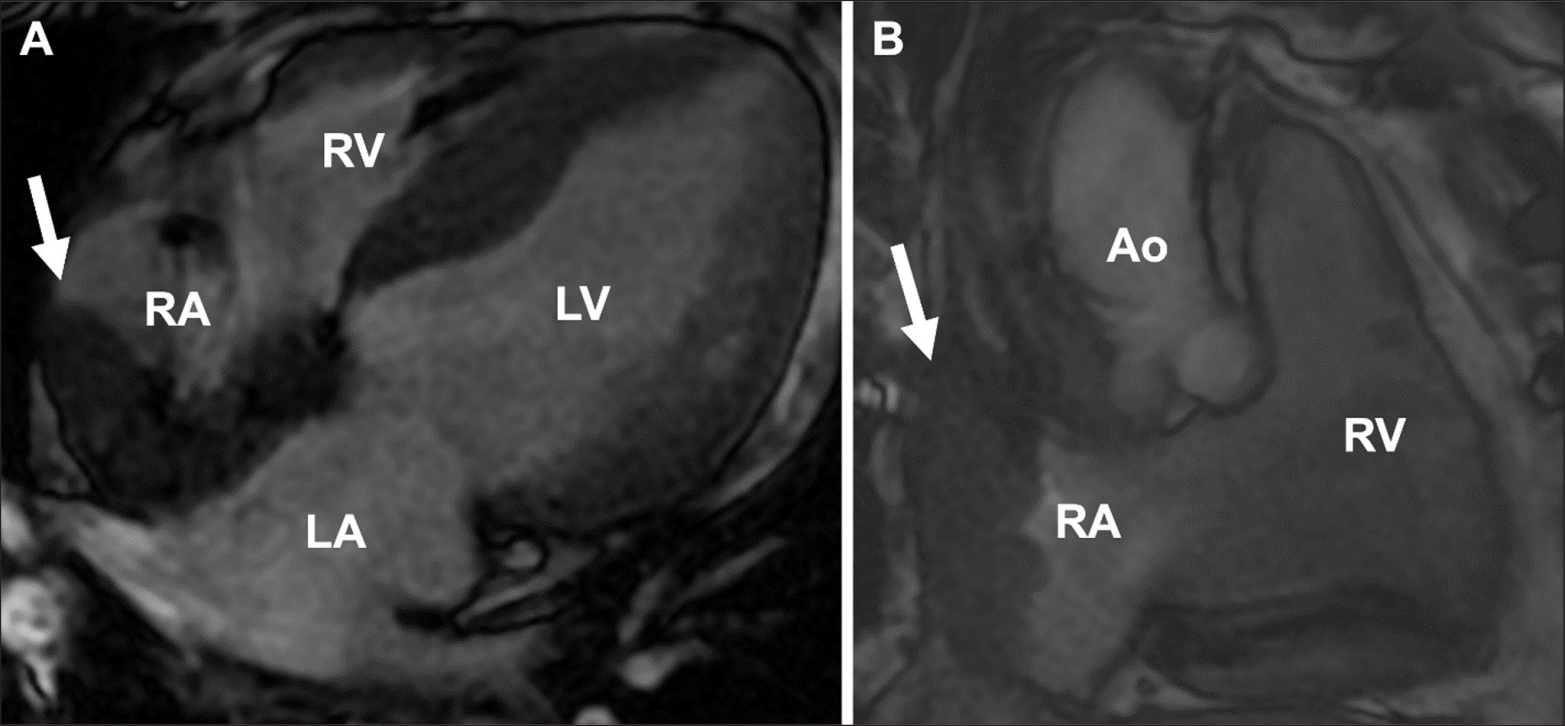
**(A)** Gadolinium cardiac magnetic resonance imaging in transverse view showing heterogeneous hyperenhancement of right atrial mass (arrow) occupying the right atrium, with right ventricle, left atrium, and left ventricle. **(B)** Same right atrial mass (arrow) redemonstrated in sagittal view with RV and aorta. RA: right atrium; RV: right ventricle; LV: left ventricle; LA: left atrium; Ao: aorta

The patient was started on empiric daptomycin for presumed endocarditis with an infected right atrial thrombus. However, blood cultures remained negative, and a transthoracic echocardiogram (TTE) did not reveal obvious vegetations of aortic, mitral, and tricuspid valves (Figure 3 A). A subsequent transesophageal echocardiogram (TEE) revealed a 4.9 cm × 4.0 cm × 2.3 cm mass in the superior vena cava and right atrium engulfing the right atrial and right ventricular pacemaker leads (Figure 3 B). Cardiac magnetic resonance imaging (CMR) with gadolinium was obtained for mass characterization, which demonstrated heterogeneous hyperenhancement suggestive of malignancy (Figure 4). Under intracardiac echocardiography guidance, a percutaneous transcatheter biopsy of the right atrial mass was performed, confirming cardiac anaplastic large-cell lymphoma. The patient was ultimately diagnosed with anaplastic lymphoma kinase (ALK) negative ALCL and commenced on six cycles of reduced-dose brentuximab combined with cyclophosphamide, doxorubicin, and prednisone. In clinic follow-up, he initially demonstrated complete metabolic response on post-treatment PET-CT (Figure 2 D). However, he later developed extra-cardiac disease relapse and eventually elected for hospice.

## Discussion

Extranodal cardiac lymphomas are extremely rare and account for approximately 1% of primary cardiac tumors and 0.5% of extranodal lymphomas.^6^ Furthermore, a case of cardiac primary ALCL is exceptionally uncommon. It is generally accepted that a primary cardiac lymphoma is a non-Hodgkin’s lymphoma if the bulk of the tumor is intrapericardial.^7^ On the other hand, metastatic cardiac involvement from systemic lymphomas is far more common (roughly 25%).^8^ Mean age of patients with primary cardiac lymphomas is approximately 60 years, with male predominance.^6,7^ The most common location is on the right side of the heart. However, cardiac lymphoma may involve any chamber and may invade adjacent structures, such as the pericardium and coronary arteries.^8^ Cardiac symptoms vary but include rapidly progressive heart failure, arrhythmias, heart block, syncope, and even restrictive cardiomyopathy. Constitutional symptoms such as fevers, chills, and night sweats are also common.

Timely diagnosis is challenging due to the rapidly progressive nature of the disease. Frequently, diagnoses are made shortly before death or at autopsy.^9^ Echocardiography is considered the first-line imaging modality for cardiac mass evaluation, particularly mobile masses. The high temporal resolution of small mobile masses makes echocardiography preferred over CT or CMR. TEE can rapidly provide information regarding size, location, mobility, and pericardial involvement of a tumor. When visualized on echocardiography, these tumors may appear as homogeneous infiltrating masses that lead to wall thickening and restrictive physiology or as nodular masses intruding into the heart chambers.^1^ However, right atrial masses can be missed on TTE because sweep maneuvers to adequately assess all aspects of cardiac chambers is not part of routine TTE scanning protocols. TEE provides higher-quality views of the entire atria, although it is an invasive modality. On CMR, tissue appears isointense on T1-weighted imaging and mildly hyperintense on T2-weighted imaging due to diffuse tissue edema.^10^ Heterogeneous enhancement may also be observed with gadolinium contrast.^11^ A PET-CT provides information on metabolism and degree of cell proliferation. The intensity of uptake is directly proportional to degree of malignancy.^12^ Following treatment, PET-CT is also performed to assess for cancer response.

Definitive diagnosis is made through biopsy or pericardial effusion analysis. Treatment is primarily with an anthracycline-based chemotherapy such as cyclophosphamide, doxorubicin, vincristine, and prednisone (CHOP regimen).^1,13^ In systemic ALCL, those who are ALK positive typically portend a more favorable prognosis than those who are ALK negative.^13^ However, it is difficult to extrapolate this to cardiac ALCL given its rarity. In general, primary cardiac lymphomas have high recurrence rates (as high as 55%) and follow an aggressive course.^14^ Prognosis of patients with primary cardiac lymphomas remain poor, as more than 60% of patients die within 2 months of diagnosis.^15^ Conducting early conversation on goals of care is an important component of the treatment plan.

## Conclusion

Although rare, cardiac lymphomas should be on the differential diagnoses of a cardiac mass in the setting of constitutional symptoms without an infectious source. A multimodality diagnostic approach is often required, and goals of care conversations should be initiated early due to the natural aggressive course of disease.
